# Health care itineraries for women in situations of abortion: methodological aspects of a qualitative study for *Birth in Brazil II* survey

**DOI:** 10.1590/0102-311XEN006223

**Published:** 2024-04-29

**Authors:** Claudia Bonan, Ana Paula dos Reis, Andreza Pereira Rodrigues, Greice Maria de Souza Menezes, Cecilia Anne McCallum, Nanda Isele Gallas Duarte, Ulla Macedo, Maiara Damasceno da Silva Santana, Débora Cecília Chaves de Oliveira, Rosa Maria Soares Madeira Domingues, Maria do Carmo Leal

**Affiliations:** 1 Instituto Nacional de Saúde da Mulher, da Criança e do Adolescente Fernandes Figueira, Fundação Oswaldo Cruz, Rio de Janeiro, Brasil.; 2 Instituto de Saúde Coletiva, Universidade Federal da Bahia, Salvador, Brasil.; 3 Escola de Enfermagem Anna Nery, Universidade Federal do Rio de Janeiro, Rio de Janeiro, Brasil.; 4 Instituto Gonçalo Moniz, Fundação Oswaldo Cruz, Salvador, Brasil.; 5 Instituto Nacional de Infectologia Evandro Chagas, Fundação Oswaldo Cruz, Rio de Janeiro, Brasil.; 6 Escola Nacional de Saúde Pública Sergio Arouca, Fundação Oswaldo Cruz, Rio de Janeiro, Brasil.

**Keywords:** Abortion, Methodology, Reproductive Rights, Women’s Health, Aborto, Metodologia, Direitos Reprodutivos, Saúde da Mulher, Aborto, Metodología, Derechos Reproductivos, Salud de la Mujer

## Abstract

In recent decades, several academic studies on abortion have been produced in Brazil, with different designs, objectives, and methodologies. However, due to the diversity of situations in which Brazilian women experience abortion, the complexity of this topic, and its modulations in different political and sociocultural contexts, it still challenges academicians and the fields of health and reproductive rights. In this article, we present methodological aspects of a qualitative study on health care itineraries of women in situations of abortion, a component of the *Birth in Brazil II* survey, whose objective is to discuss the effects of gender; race/ethnicity; social class; generational, regional, and territorial inequalities on care itineraries. We discuss the study design development, the construction of the theoretical framework and specific analytical axes, the development of interview instrument, definition of participant selection criteria, strategies to contact participants and conduct the interviews, management of field work and materials produced, analytical procedures, and ethical issues. In total, 120 narrative interviews were conducted in order to include a diversity of women and obtain detailed results from the quantitative analysis under *Birth in Brazil II* survey. The context of criminalization of abortion has an impact on the production of knowledge on this subject, creating challenges such as difficult access to women, women’s anonymity, privacy and data confidentiality, creation of objective and subjective conditions so that they can narrate their experiences in depth. With this article, we seek to contribute to the debate about these challenges in abortion research in Brazil.

## Abstract (pt)

Nas últimas décadas, produziu-se um robusto corpus de pesquisas sobre aborto no Brasil, com diferentes desenhos, objetos e metodologias. Contudo, pela diversidade de situações em que as mulheres brasileiras vivenciam o abortamento, pela complexidade do tema e por suas modulações em contextos políticos e socioculturais distintos, o assunto não cessa de desafiar a academia, o campo da saúde e dos direitos reprodutivos. Neste artigo, apresentamos aspectos metodológicos de um estudo qualitativo sobre itinerários de cuidado à saúde de mulheres em situações de abortamento, componente da pesquisa *Nascer no Brasil II*, que objetiva discutir efeitos das desigualdades de gênero, de raça/etnia, de classe social, geracionais, regionais e territoriais nesses percursos. Discutimos o desenvolvimento do desenho do estudo; a construção do arcabouço teórico e recortes analíticos específicos; a elaboração do instrumento de entrevista; os critérios de seleção das mulheres; as estratégias de abordagem e condução das entrevistas; a gestão do fluxo do campo e dos materiais produzidos; os procedimentos analíticos; e os problemas éticos. Para incluir uma diversidade de mulheres e aprofundar resultados do componente quantitativo do *Nascer no Brasil II*, serão realizadas 120 entrevistas narrativas. O contexto de criminalização do aborto impacta a produção de conhecimento sobre o tema, impondo desafios como conseguir acesso às mulheres, assegurar o anonimato e sua privacidade, além do sigilo das informações, gerar condições objetivas e subjetivas para que possam narrar em profundidade as suas experiências. Com este artigo, procuramos contribuir para o debate sobre esses desafios das pesquisas sobre aborto no Brasil.

## Abstract (es)

En las últimas décadas, se produjo un robusto corpus de investigaciones sobre el aborto en Brasil, con diferentes diseños, objetos y metodologías. Sin embargo, debido a la diversidad de situaciones en las que las mujeres brasileñas vivencian el abortamiento, la complejidad del tema y sus modulaciones en diferentes contextos políticos y socioculturales, el tema continúa desafiando a la academia, el campo de la salud y los derechos reproductivos. En este artículo, presentamos aspectos metodológicos de un estudio cualitativo sobre los itinerarios de cuidados de la salud de mujeres en situación de abortamiento, componente de la encuesta *Nacer en Brasil II*, que tiene como objetivo discutir los efectos de las desigualdades de género, raza/etnia, clase social, generacionales, regionales y territoriales en esos recorridos. Discutimos el desarrollo del diseño del estudio, la construcción del marco teórico y los recortes analíticos específicos, la elaboración del instrumento de entrevista, los criterios de selección de las mujeres, las estrategias de abordaje y realización de las entrevistas, el manejo del flujo del campo y de los materiales producidos, los procedimientos analíticos y los problemas éticos. Para abarcar una diversidad de mujeres y profundizar los resultados del componente cuantitativo de *Nacer en Brasil II*, se realizarán 120 entrevistas narrativas. El contexto de criminalización del aborto impacta la producción de conocimiento sobre el tema, imponiendo desafíos, tales como conseguir acceso a las mujeres, asegurar su anonimato y privacidad y la confidencialidad de la información, generar condiciones objetivas y subjetivas para que puedan narrar en profundidad sus experiencias. Con este artículo buscamos contribuir al debate sobre estos desafíos de las investigaciones sobre el aborto en Brasil.

## Introduction

In Brazil, abortion laws are restricted - pregnancy termination is allowed only when there is a risk to woman’s life, when the pregnancy results from rape, and in case of fetal anencephaly. However, induced abortion is often performed, mostly in unsafe conditions. It is an important factor of maternal morbidity and mortality ^1^ ^,^ ^2^, with studies indicating it accounts for 5% of all hospitalizations of women of reproductive age ^3^. For this reason, it is considered a public health problem. However, spontaneous, induced, and legal abortion is also a problem of reproductive rights and social justice, given the stigma and discrimination women have to face, also in health services ^4^ ^,^ ^5^ ^,^ ^6^, and the way it affects, in particular, poor, black, and periphery women ^7^.

We have a relevant number of academic studies on abortion in Brazil, with different designs, methodologies, and subjects analyzed. A review of the Brazilian literature on the subject, between 1987 and 2007, surveyed 2,109 publications and identified 398 original studies, with empirical data on the profile of women who have abortions, their abortion trajectories, abortion in adolescence, complications and sequelae of induced abortion, the use of misoprostol (medication to terminate pregnancy), among others ^8^. The review also identified gaps in the academic production, such as scarce studies on spontaneous abortion, legal abortion, abortion due to fetal malformation, and abortion in the private sector, few empirical investigations outside the Southeast Region, in addition to poor discussion of issues such as race and disability. In 2007, the scientific literature on abortion started to expand, with the development of several studies ^2^ ^,^ ^5^ ^,^ ^6^ ^,^ ^7^, but many of the gaps highlighted in the review study ^8^ are still observed.

In order to contribute to studies on abortion, the study *Cuidado à Saúde de Mulheres em Processo de Abortamento e a (re)Produção da (in)Justiça Reprodutiva no Brasil* (Health Care for Women Undergoing an Abortion and the (re)Production of Reproductive (in)Justice in Brazil) was conducted in 2019 as a qualitative study under the *Birth in Brazil II: National Research on Abortion, Labor and Childbirth* (2021 to 2023) (*Birth in Brazil II*). Unlike the first version of this research project, the *Birth in Brazil II* included in its objectives an evaluation of care for hospitalized women with a diagnosis of abortion. It identified knowledge should be expanded to understand how women experience these events that are part of their reproductive lives and the conditions in which care and self-care practices occur in these situations.

It resulted in the development of a questionnaire applied to women admitted to hospitals/maternity wards - the first stage of the *Birth in Brazil II* survey - which includes questions about abortion, with two sections exclusively dedicated to these cases. Also, the *Birth in Brazil II* project incorporated a proposal for a qualitative study to analyze the experiences and health care practices of women with abortion, based on their own perspectives.

In this study, abortion refers to different situations that include at least four events: spontaneous abortion (including hydatidiform mole), induced abortion, legal abortion (including risk to the pregnant woman’s life, pregnancy resulting from rape, and fetal anencephaly), and ectopic pregnancy. Each of these situations involves many medical, sociological, and legal specificities that affect the context of care and should be investigated. However, in a country where abortion is heavily criminalized, stigmatized, and ideologized, all women who have had an abortion are, to some extent, vulnerable to mistreatment, neglect, moral harassment, and denial of care, and are exposed to risks that affect their safety, privacy, health, and even their lives.

It does not mean equal distribution of vulnerabilities and exposure to risks. In addition to the vulnerabilities and risks inherent to clandestine abortion and even to legal abortion - given the repression of conservative groups against women and health professionals, difficult access to services, and abuse of conscientious objection by physicians - gender, ethnic, racial, social class inequalities, among others, deeply mark the experiences of women who have spontaneous abortion or voluntary pregnancy termination, affecting their self-care and care conditions in health services, producing and reproducing reproductive injustice ^2^ ^,^ ^5^ ^,^ ^6^.

The theoretical idea of this study is based on the issue of health care practices for women linked with abortion and deficits and inequalities in the distribution of reproductive justice. Reproductive justice refers to the idea that exercising reproductive rights and reproductive autonomy is prohibited when sexuality and reproduction are experienced in situations involving economic, social, political injustice and racist, ethnic, homophobic, transphobic, sexist, classist discrimination and violence, among other factors ^9^ ^,^ ^10^. With this notion, we intend to use an analytical axis that addresses the intersectional forms of oppression that threaten the rights, bodily integrity, and autonomy of certain groups of subjects, more than others.

Based on the issues above, we conducted a *Qualitative Study on Abortion* under the *Birth in Brazil II* to analyze the health care itineraries of women in situations of abortion with different reproductive histories; sociocultural contexts; and sociodemographic, affective-sexual, and family profiles. Our general objective is to understand the effects of gender; race/ethnicity; social class; generational and regional inequalities on care itineraries, focusing on relationships with health services and professionals and relationships with other subjects and groups. This study will produce original analyses on the experiences of women from the five regions of Brazil, hospitalized in private, public and mixed health care system hospitals, filling important gaps in the academic literature on this topic.

In this article, we present methodological paths of the qualitative study, including theoretical, analytical, operational, and ethical particularities.

## Development of study design

The study design to assess health care itineraries was inspired by an intersection of methods to analyze therapeutic itineraries ^11^ ^,^ ^12^, life histories, and narratives ^13^ ^,^ ^14^ ^,^ ^15^. Based on women’s reports obtained through narrative interviews ^14^, we sought to understand journeys in search of therapeutic care, sociocultural health practices and the meanings given to their experiences, both inside and outside health services, as well as the contexts and structures of power inequalities that surround them.

Care is a category that links practice and value, work and ethics ^16^ ^,^ ^17^. Practice-value care involves social relations of people, communities, and institutions and are produced and performed in specific historical contexts, with their own norms and power dynamics. Care relationships imply providing a subject, groups of subjects or larger communities with the best conditions of safety, well-being, development and physical, psycho-emotional, moral, and material integrity ^18^. Care economy ^18^ -social organization of care production, distribution, and consumption - respects the preponderant values of the specific contexts where it is organized, and can be based on community, market, and/or democratic foundations ^19^ ^,^ ^20^ ^,^ ^21^ and, in societies structured by class, race, and gender hierarchies, it determines social inequalities.

Based on the analysis of care itineraries, this study aims to assess the care relationships involved in the journey of women with abortion, discussing how in a scenario of criminalization, punishment, and stigma, care relationships tend to be fragile and, in certain circumstances, may become the opposite: neglect, mistreatment, violence, torture, and death.

As a component of *Birth in Brazil II* research, this Qualitative Study on Abortion is aligned with the questions and protocol of the core research, published in Leal et al. ^22^. In few words, *Birth in Brazil II* is a national hospital-based survey with a planned sample of 22,050 people hospitalized for childbirth and around 2,205 for abortion care in 465 health facilities that perform 100 or more births every year. The sample was stratified by macro region of the country (North, Northeast, Southeast, South, Central-West), type of hospital (public/private/mixed), and location (capital and municipalities in the Metropolitan Region/non-Metropolitan Region).

In each hospital, postpartum women, whether cisgender women or transgender men, of any age who had live births or fetal deaths admitted with a diagnosis of abortion identified in hospital records were considered eligible. Women who gave birth in another institution, at home or in a public place, who did not speak Portuguese, with severe hearing or mental disability, and those hospitalized for delivery by court order were considered ineligible.

For all *Birth in Brazil II* participants, three interviews are planned in the quantitative analysis, in addition to collection of information from prenatal cards and hospital records. The first is conducted in person during hospital stay. Later, two telephone interviews are performed 2 and 4 months after birth/abortion to assess the use of services after discharge, late maternal and neonatal morbidity, breastfeeding, mental health, and mistreatment in childbirth and abortion care.

This Qualitative Study on Abortion has no other general inclusion and exclusion criteria other than the hospital survey. However, the development of theoretical problems and research questions to be analyzed in this stage implied the selection of specific analytical axes and criteria for the creation of heterogeneous universes of women with specific experiences and/or situations who share fetal loss/abortion. So far, participants interviewed in the qualitative stage of the research have been cisgender women. Therefore, we chose to use the term “woman” or “women” in this article; however, we were aware of the importance of ensuring visibility to different gender identities in research analyses and publications.

The review of scientific literature on abortion in Brazil included searches in national and international databases. Due to their specificities, we initially categorized the searches into themes such as: spontaneous, induced, and legal abortion. Then, using questions related to the literature analyzed, we developed the following hypotheses to be investigated: (1) in our context, all forms of abortion, to some extent, involve stigmas, moral judgments, ideological and criminal repression; (2) vulnerabilities and risks of women with spontaneous, induced, legal abortion or fetal loss due to ectopic pregnancy are not randomly distributed, but reproduce cleavages of social markers of differences in class, race, territory, generation, among other factors.

First, we established the specificities of the care itineraries as analytical axes according to type of abortion, race/color, age/generation, region of the country, residence in large urban centers or smaller cities and rural territories, and hospitalization in public or private service.

The literature review showed a lack of studies on care itineraries in some specific situations, such as the experience of severe maternal morbidity or near miss resulting from abortion. Then it was also selected as a specific axis of analysis. In addition, the beginning of the study was affected by the new coronavirus pandemic so scientific publications addressed its risks and consequences for pregnant women ^23^, which encouraged specific analysis of care itineraries of women reporting COVID-19 during pregnancy.

All these analytical axes lead the selection of eligible women from the *Birth in Brazil II* database and the creation of a heterogeneous universe of participants, paying attention to groups that are not commonly investigated, such as adolescents, indigenous people, residents of the North Region and rural areas, women treated in private services, women who had COVID-19 during pregnancy, women with serious maternal morbidities or near miss as a result of the abortion, and women who reported induced or legal abortion or ectopic pregnancy.

Narrative interview was the technique used in this study to analyze care itineraries. Since the project was developed before the COVID-19 pandemic, qualitative interviews were planned by telephone or digital system and recorded with the consent of the interviewee, a strategy that has been suitable for the investigation of sensitive topics such as abortion. It helped us overcome the effects of the pandemic on studies that involve face-to-face interviews.

The qualitative interview instrument was developed according to some criteria. First, it should meet the needs of the narrative interview, i.e., avoid the question-answer scheme and propose themes to encourage the interviewee to freely narrate her story, select and order events, address the topics on her own way, contextualize the topics, make correlations, and ensure meanings to her experience ^14^. Second, although the instrument proposed major questions to all interviewees, it should be used in a flexible manner to address aspects from each of the specific axes. Therefore, this instrument had four sections: experience during hospitalization due to abortion; care itinerary before arrival at the hospital; the context of life and pregnancy; synthesis of the most significant aspects of the experience of becoming pregnant and having an abortion(Supplementary Material - Figure S1: https://cadernos.ensp.fiocruz.br/static//arquivo/suppl-e00006223_2992.pdf).

The field work began in March 2022 and is scheduled to end in December 2023. In order to cover a diversity of situations and profiles of interviewees and gather enough material to discuss the various specific analytical axes, 120 interviews were scheduled for this qualitative study.

## Technical and operational strategies and procedures of the study

After defining the analytical axes and developing the qualitative interview instrument, we established the field work flow. The qualitative study process had to be adjusted to the times and strategies of the quantitative study, which, as seen above, had three interviews with each participant: (1) the first interview conducted in person during hospital admission using a structured questionnaire to collect sociodemographic, reproductive, obstetric, and clinical information; (2) the second interview conducted via telephone two months after birth or abortion, using a questionnaire to analyze the prevalence of maternal and neonatal morbidity; use of postnatal health services; satisfaction with care; and symptoms of depression, anxiety and post-traumatic stress disorder (telephone interview 1); and (3) the third interview (second via telephone interview) conducted four months after birth or abortion, using a questionnaire to investigate the occurrence of mistreatment in the care received, satisfaction with care, discrimination in everyday life and breastfeeding (telephone interview 2). Together with the *Birth in Brazil II* survey team, we established an ideal interval between the qualitative interviews about abortion, preferably between telephone interviews 1 and 2.

To conduct the interviews, we organized a flow and developed a spreadsheet for the addition of the following information: codes of interviewees in the hospital stage; information about the state of origin, race/color/ethnicity, area of residence (urban/rural), hospitalization in a public/private/mixed service; whether contact was made and whether the woman agreed with telephone interview 1; minimum and maximum date for the qualitative interview; whether contact for telephone interview 2 has been made; thematic group(s) (specific axes) in which the interviewee fits; name of the designated interviewer; history of contacts and attempts to schedule the interview; date of interview or refusal/withdrawal; control of transcriptions. A color scheme highlights potential interviewees not yet contacted, contacts in progress, interviews conducted, contact refusal or removal by the team, telephone numbers that do not exist or belong to someone else.

This spreadsheet is fed by the *Birth in Brazil II* database, which is stored on the REDCap platform (https://redcap.fiocruz.br/redcap/), and by the qualitative study researchers, who regularly generate reports with a balance of this flow. It also provides an access link to information on the REDCap platform obtained from the questionnaires applied in the previous stages, which supported the selection of participants and singularization of interviews.

First, we conducted a pilot study in which we tested types of approaches, interviews, and the instrument, seeking to improve and develop techniques that were sensitive to the heterogeneity of the participants. Qualitative interviews were conducted via traditional telephone call, WhatsApp or another messaging app and, in some cases, via video call.

In the first interviews, some challenges were observed: non-existent telephone numbers or numbers belonging to other people, participant’s difficult access to internet, lack of privacy and/or free time to provide a longer interview due to work outside the home, household chores and/or childcare. To overcome barriers to study participation, we sought to diversify the repertoire of approach strategies - including messages via app and telephone calls, at different times and on different days - and interview scheduling, offering a variety of times and days, conducting interviews on two or three different days, as required. Regarding the type of interview, when a telephone conversation was not possible, the interview was conducted via messaging.

The fact that the *Birth in Brazil II* has several stages required special attention. In order to remind participants that the research continued after the initial interview, explain the characteristics of the qualitative interview, and highlight its importance, we created the following text that supports the initial contact via messaging app:

“*Hello, how are you,* [name of participant]*? I am* [name of interviewer], *from the Birth in Brazil research team, in which you agreed to participate at the* [hospital name]. *My colleagues have already spoken to you in previous phases - in the hospital and on the phone, remember? I am part of a new phase of the research, so I am contacting you now to talk a little more, but this time I want to hear you speak with your own words in a free conversation. For us, it is very important to know what you have to say. Is it possible? We can schedule another call when you can talk*”.

The proposal for a free conversation had different reactions, sometimes seen as a relevant part of the study, as many potential participants want to talk more and freely about their abortion experience, or sometimes as a nuisance, with some participants reporting they had “already said it all”. In this case, it is necessary to understand whether this is a refusal to study participation or just to the free conversation, and if they are only reluctant to what they see as one more questionnaire, indicating temporary unavailability or a hesitant attitude towards the research. To exhaust the possibilities to conduct the interview, but in a respectful manner, negotiations are conducted without pressure and, sometimes, over a long period of time. It means that, in many cases, the qualitative interview is scheduled only after telephone interview 2, a change that required a new conversation with the quantitative study teams to rearrange the flow.

The criterion to contact potential interviewees only after the first quantitative telephone interview also had to be more flexible, because for a significant number of women who were part of our priority groups, contact at 2 months after hospital discharge had not been successful. Specifically for these women, an additional process was created, which consisted of making a new contact, always respecting their attitude of not wanting to continue in the study.

Lack of response to contacts, hesitation, and refusals should also be interpreted as a component of the challenges found in studies on abortion in Brazil. Silence and secret surrounding the abortion experience ^24^ ^,^ ^25^ are expressions of a context that articulates the weight of legal prohibition, stigmas, and cultural and moral taboos ^26^. In order to minimize these problems, the team was trained to build a welcoming and non-judgmental dialogue.

In this sense, language related to the abortion experience had special attention. Interviewers were instructed not to anticipate or presuppose the relationship between abortion and suffering/trauma, and to remain open to expressions of suffering regarding abortion. This concern is based on findings from several studies that identified diversity, ambivalence or ambiguity, polyphony or multifaceted awareness of narratives about abortion ^27^ ^,^ ^28^ ^,^ ^29^ ^,^ ^30^ ^,^ ^31^. Therefore, especially during the interviews, respect for such multiple possibilities of giving meanings to the experience with spontaneous or induced abortion is also built through vocabulary mirroring techniques. Interviewers avoid naming this experience, in its physiological, cultural and emotional aspects, using the vocabulary adopted by the interviewees.

Finally, field work experience indicates that most participants want to share their pregnancy and abortion stories. Interviews often last more than an hour and some cases require more than one meeting to complete their stories. The different scenarios where women are when they are interviewed, whether by telephone or video calls, are also highlighted during the conversation, including sounds all around, such as noises from the television in the background, household appliances, work tasks, voices of children and family members, among others. Interruptions due to household chores and professional tasks are also part of the interview. In order to record these data, interviewers are encouraged to produce summaries of their impressions and descriptions of the interview context, which are attached to the qualitative field material.

With the participant’s oral consent, the interviews are recorded, shared only on secure platforms, and transcribed, with names and other personal information suppressed.

Then, the field work flow of the qualitative stage was consolidated as follows:

(1) Biweekly update of the spreadsheet of eligible women for qualitative interviews, as new cases are registered in the *Birth in Brazil II* database with a coded scheme and restricted access;

(2) Distribution of contacts to interviewers according to selection criteria, with thematic priority markers;

(3) The interviewers sent a standardized initial message via WhatsApp to women who left contact or contacted them through other social media or initial phone call;

(4) Continuity of contact using different strategies depending on the answer (or in the absence of an answer);

(5) Interview schedule, recording refusal or maintaining an open dialogue when there is uncertainty or no response;

(6) Reevaluation of cases that remain “open” for a long period, changing interviewers for new attempts;

(7) When the woman is willing to provide an interview: offering different interview methods (video call, audio call, telephone call, or if these options are not possible or desired, continue the interview via messaging), and different times or days of the week. If the interviewer is unavailable, the team will replace her;

(8) Preparation for the interview, reading of the thematic script and the questionnaire answered by the participant in the maternity ward; preparation of the device to make the call and device positioning for audio recording;

(9) Start of the interview with an explanation of study objectives, the participant’s rights, and request for consent for audio recording;

(10) Writing of a summary with field impressions immediately after the interview;

(11) Safe storage of audios and transcriptions with anonymity;

(12) Gathering of transcriptions, summaries, and field impressions to constitute the qualitative database for analysis.

Regarding the analysis techniques, the following used in combination: narrative analysis and thematic categorical analysis. For Jovchelovitch & Bauer ^14^ (p. 91), in the act of narrating “*people remember what happened, put the experience in a sequence, find possible explanations for it, and arrange the chain of events that build individual and social life*”. According to these authors, the structure of a narrative is made up of a number of events triggered by the person who narrates, ordered in a certain sequence: presentation of the context where things happen and the actors involved in the events narrated; motivations for the actions of the narrator and other people and their relationships and interactions; causal associations; value judgments about events and evaluation of their effects or results. However, these components are individualized only for analytical purposes, because the narrative is a whole that configures a plot, and within this plot the meaning of the narrated experiences is produced.

In turn, the thematic categorical content analysis enhances the narrative analysis because, according to Minayo ^32^ (p. 209), it aims to “*discover the cores of meaning that make up a communication whose presence or frequency mean something for the analytical objective sought*”.

These analytical matrices guide our study. In the stories told about abortion, we seek to identify the narrative components, scrutinize the care relationships created in the course of events, and interpret the meanings that surround these experiences.

The stages of the analytical work performed by the team are inspired by the proposals of Bardin ^33^, such as: (1) individual floating reading, followed by presentation and collective discussion of the interviews; (2) identification of themes and categories of narratives, systematization in a spreadsheet and discussion of these thematic categories based on the literature on the topic; and (3) interpretation of narrated experiences, construction of meaning cores and discussion, taking into account questions, objectives, and theoretical problems of the study.

## Parameters and ethical considerations

As part of the *Birth in Brazil II* survey, this qualitative study was approved by the Brazilian National Research Ethics Committee (CONEP) on March 11, 2020 (CAAE 21633519.5.0000.5240) and by local research ethics committees of the participating institutions, or the clinical directors when local committees were absent. The study strictly respects the ethical regulations of CONEP *Resolutions n. 466/2012* and *n. 510/2016*. All participants sign an informed consent form (ICF) and, for adolescents, the consent form was signed by legal guardians in the hospital stage of the study.

However, the ethical-methodological aspects of abortion studies involve specific challenges, which have been widely discussed in Brazil ^5^ and include problems in safe access to data and first-person accounts, protection of this information, and guarantee of participant confidentiality and anonymity. Considering the experience of the *Birth in Brazil II* team with reproductive rights and justice, these challenges implied ethical-political commitments, with the development of strategies and pacts among researchers.

Contact with potential interviewees for the study is made in the hospital by the hospital survey team. At this moment, information is provided about the study objectives, methodology, risks, and benefits and, if agreed to be interviewed, a consent form is signed. Later, when contacting women to invite and schedule a qualitative interview, the interviewers only mention the hospital admission, without characterizing it as an abortion, preventing third parties from reading sensitive messages. At this point, the researchers reiterate that granting the interview is voluntary, that anonymity and confidentiality are guaranteed, and that refusal to answer any question or withdrawal from participation is permitted.

Audios and transcriptions of interviews are stored with the *Birth in Brazil II* materials on the REDCap platform, hosted on a Oswaldo Cruz Foundation (Fiocruz) server. Access to the platform is restricted to executive coordination and all interviewers of the team sign a responsibility agreement to ensure participant anonymity and data confidentiality. For analysis of the material and subsequent publications, fictitious names are used and descriptors that could identify people are discarded.

In addition to data protection, participant confidentiality and anonymity, an important ethical consideration in abortion studies is the establishment of the interview as a welcoming space for women to share their stories without judgment and stigma. It has been a central methodological concern. We include strategies of attention to sensitive situations such as requests for help, demonstration of suffering, and identification of situations of violence. In these cases, referrals are discussed with the research coordination, using protocols established by the *Birth in Brazil II*, such as provision of information about mental health services and how to access services of protection against violence and psychosocial support in the participant’s region.

## The research team: interdisciplinary and political-academic background

The research is developed by a multidisciplinary, intergenerational team from two regions of the country (Northeast and Southeast). The study was initially conceived by five researchers with different disciplinary backgrounds (social sciences, communication, nursing, and medicine) and academic experience in reproductive and sexual health, gender and health studies and, in particular, public health. The political-activist dimension that gathers the team members should also be mentioned, as they all define themselves as feminists, anti-racists, and engaged in the fight for sexual and reproductive rights and strengthening of the Brazilian Unified National Health System (SUS). This dimension has particular importance as it is directly related to how the research object is framed and interpreted. Between 2021 and 2022, the team received new members with similar training to the original researchers.

The project started during the first months of the COVID-19 pandemic and its development has been almost entirely offline. The study implementation encouraged the creation of the *Gender, Reproduction and Justice* (RepGen) research group, which is now involved in other scientific investigations.

## Final considerations

Focusing on the health care itineraries of women in situations of abortion, participants in the *Birth in Brazil II* survey, the qualitative stage methodology was developed to consider the diversity of reproductive stories, sociocultural contexts, sociodemographic profiles, and relational dynamics. The perspective of reproductive justice is present in the development of all strategies - theoretical focus, elaboration of research problems, development of the instrument and field construction procedures - and deeply inspires the ethical aspects and care adopted. Using analysis that articulates narrative and thematic content, we intend to understand how the experiences of women with spontaneous or induced abortion are marked by gender, ethnic-racial, social class, generation, and existential territory inequalities, among others, and the impact of these relationships on their self-care and care conditions and, consequently, on their lives.

The context of criminalization that surrounds abortion in Brazil - whose effects are also seen in situations of legal or spontaneous abortion - involves challenges and affects the production of knowledge on this topic ^5^. It is particularly challenging to encourage women to participate and talk about their abortion experiences through telephone interviews - a relatively new strategy in Brazil and opportune in the context of the COVID-19 health crisis. This data production tool is common in global north countries, especially in investigations addressing delicate or sensitive topics ^34^ ^,^ ^35^. By avoiding face-to-face interaction between interviewees and interviewers, the telephone strategy can favor narratives about stigmatized or difficult-to-declare events, as in this study. In the case of induced abortion, in particular, the impact of political situations in recent years cannot be minimized as they do not favor debates on issues such as reproductive rights and abortion, inhibiting women from talking about their experiences, also beyond their intimate circles. Another challenge that represents study limitations refers to social inequities that are also expressed as digital inequalities, marked by different domains of access to and abilities to use these technologies at the individual and macro level, which include life course, gender, race and class, economic activity, and social capital ^36^. The search to mitigate these inequalities finds, in the initial face-to-face approach at the hospital stage, an ally in the integration of analogue and digital strategies towards a diversification of participant profiles. Methodological aspects are thus intertwined with ethical care, adopted by a research team that is trained to listen respectfully and without stigmas.

The incorporation of an evaluation of care for women with abortion in the second *Birth in Brazil* survey, as well as the inclusion of a qualitative component for this group, materializes the recognition of events related to abortion as an inseparable part of a public policy agenda that ensures full practice of women’s sexual and reproductive rights, addressing structural inequalities in the context of their sexuality and reproduction experiences and promoting the construction of reproductive justice.
