# Supplementary material for: Health care itineraries for women in situations of abortion: methodological aspects of a qualitative study for Birth in Brazil II survey
Source: Cad Saude Publica. 2024 Apr 29;40(4):e00006223. doi: 10.1590/0102-311XEN006223 (PMC11057479; doi:10.1590/0102-311XEN006223)
Supplement: Supplementary file 1 [file 1678-4464-csp-40-04-PT006223-s.pdf]

Material suplementar

Figura S1 Instrumento-guia para entrevista narrativa.

ESTUDO QUALITATIVO SOBRE ABORTO DA PESQUISA NASCER DO BRASIL II:
ITINERARIOS DE CUIDADOS À SAÚDE DE MULHERES EM SITUAÇÕES DE ABORTAMENTO

Instrumento-guia para entrevista narrativa

CONHECENDO A PARTICIPANTE

Pergunta disparadora:
Você pode se apresentar? Por favor, me conte um pouco sobre você, sua vida, seu dia-a-dia, seus sonhos.

Bloco comum a todas

Idade | Raça/etnia | Religiosidade/espiritualidade/crença | Sexualidade, conjugalidade, parceria afetiva | Filhos | Estudos ou trabalho profissional |Trabalho doméstico e/ou cuidadode de outras pessoas | Rede: amigos/familiares | Território de moradia | Rotinas e estratégias de autocuidado | Uso de serviços de saúde | Projetos de vida/aspirações

EXPERIÊNCIA HOSPITALAR

Pergunta disparadora:
Eu gostaria agora de conversar sobre o tempo que a você ficou internada no [hospital/maternidade X]. Como foi? Como foi seu atendimento?

Bloco comum a todas

Percurso de cuidado na instituição (tempo de espera, fluxos de atendimento, setores onde passou)
Primeiro atendimento (setor específico, se era identificado, privacidade)
Cuidado pelos profissionais (forma de tratamento, comunicação, empatia, respeito, maus-tratos, privacidade)
Ambiência (limpeza/aeração/conforto)
Informações recebidas (Se sentiu bem informada? Ficou com dúvidas? Pode fazer perguntas?)
Exames realizados (laboratoriais, USG, ISTs, tempo de espera para realizar)
Internação (ambiente, privacidade, relações com as demais mulheres
Direito a **acompanhante e** presença do **parceiro**
Tipo de procedimento realizado (preparação, analgesia, informações ou possibilidade de escolha do procedimento medicamentoso, amiu ou curetagem)
Informações recebidas e/ou solicitadas sobre **restos fetais**, exames e outros encaminhamentos relacionados
Oferta de **métodos contraceptivos** (se vieram acompanhados de informações sobre planejamento familiar)
Alta hospitalar (indicação de revisão, consulta para contracepção, resguardo/puerpério, outros)

Atenção aos eixos transversais: estigma, discriminações, violência obstétrica / maus-tratos físicos ou verbais, desrespeito (suspeição do que fala e de seus saberes),crenças religiosas presentes nas relações de cuidado

+ Questões de atenção adicional por blocos:

Aborto legal Desconfiança nos serviços / Tentativas de demover da decisão /Resistência da equipe ou do profissional em realizar o procedimento / Espera prolongada / Objeção de consciência
Aborto espontâneo Desconfiança do profissional /acolhimento no atendimento / expectativas de ter "resposta"sobre a causa da perda e/ou de fazer um"tratamento" para problema relacionado à perda
Near miss e Morbidade Materna Grave Experiência de internação UTI
Aborto provocado: ameaça de denúncia / punição através de técnicas (ex. USG visualizar o feto/ negação do cuidado e/ou retardo na realização de procedimentos /manejo da dor

ITINERÁRIO DE CUIDADOS (da ocorrência/decisão do aborto à busca pelo hospital)

Pergunta disparadora:
Eu gostaria de conversar sobre o que aconteceu antes de você ir para o serviço/hospital, desde o momento em que você começou a abortar/decidiu interromper

Bloco comum a todas

Pessoas, locais, redes, instituições, sentimentos

Acessibilidade, informações e disponibilidade de serviços (antes edurante o abortamento) / Parceiros / Redes de apoio (família, vizinhança, amigos, instituições e representantes religiosos, redes feministas presenciais e virtuais, profissionais solidários) / Pandemia impactando no itinerário

+ Questões de atenção adicional por blocos:

Abortamento legal
Conhecimento prévio sobre o direito ao aborto legal
Tempo entre a decisão e a realização (entre a demanda/ autorização pela justiça, dificuldades com a justiça)
Relação com as questões judiciais
Sentimentos envolvidos na decisão

Abortamento espontâneo
Percepção sobre início do abortamento
Sentimentos envolvidos
Motivação e percurso de ida ao hospital

Abortamento provocado
Sentimentos envolvidos no processo
Motivação, decisão e sentimentos sobre a ida para o hospital
Percurso realizado
Impactos da criminalização na busca por atenção a intercorrências (receios, antecipação de estigma, adiamento da busca por ajuda)

Near miss e Morbidade Materna Grave
Percepção de gravidade antes da chegada ao hospital
Perda de consciência / outros sinais físicos de gravidade
Contou com antes da chegada no hospital diante da gravidade?

CONTEXTO DE ENGRAVIDAMENTO

Pergunta disparadora:
Eu gostaria agora de conversar sobre sua situação e sua vida quando você descobriu que estava grávida.

Bloco comum a todas

Uso de **método** contraceptivo (planejamento de filhos) **Constatação** da gravidez (como, quando/tempo de gestação/atraso menstrual, onde e reação) **Exames** para confirmar: exame de farmácia, exame laboratorial e USG) | **Desejo/planejamento** da gravidez | Compartilhamento da **notícia e reações** dos interlocutores | Situação de **violência sexual** no engravidamento | **Parceiro** (situação da relação, parceria, moravam juntos, participação na decisão)| **Redes** de apoio (família, vizinhança,amigos, redes feministas presenciais e virtuais) | **Estudos e trabalho** | Situação de **moradia** | **Pandemia** impactando na decisão

+ Questões de atenção adicional por blocos:

Abortamento legal
Decisão /Fontes de informação/
Processo de reconhecimento da legalidade do procedimento (risco de vida, anencefalia e violação sexual; outra não prevista na lei)

Abortamento espontâneo
Experiências anteriores de aborto
Dificuldade de engravidar, tratamento para engravidar
Cogitava o desfecho/Pré-natal /Sinais da perda

Abortamento provocado
Decisão
Método utilizado (como soube, como conseguiu, como usou ou como acessou, custos, apoios no processo)
Manejo (estratégias, dia e local de escolha)
Tempo entre a decisão e a realização
Alguém tentou impedir que levasse a interrupção adiante? Quem/Por quê?
Processo de abortamento (o que saiu/como ela a nomeia/ o que sentiu física e emocionalmente);
Definição do momento de procurar o hospital e forma de contar quando chega ao serviço
Opiniões sobre o aborto

SÍNTESE

Pergunta disparadora:
De tudo o que conversamos nesta entrevista, gostaria de saber o que mais marcou você nesta experiência. Tem algo mais que você que queira acrescentar?

Bloco comum a todas

Marcos da experiência | **Experiência prévia** semelhante/comparação | O que **ouviu** de algum profissional quetenha ficado marcado (**vínculo/estranhamento**) | **Comportamento** durante a internação e relação com a **formacomo é tratada** a mulher em situação de abortamento | Presenciou alguma cena de maus-tratos ou descaso com**outras mulheres** na mesma situação | **Significado** do evento/perda | **Situação de proibição** do aborto e aspráticas pelas mulheres (mudaria a trajetória, a forma de contar quando chega ao serviço ou com as pessoas) |Moralidades **religiosas** envolvidas no processo | **Mudanças** na vida/ relacionamentos depois da experiência. | Expectativas ou não sobre uma próxima gestação

+ Questões de atenção adicional por blocos:

Abortamento legal ou abortamento provocado
Se encontrasse/conhecesse/descobrisse futuramente alguém vivenciando a mesma situação, ajudaria com informações/aprendizados do seu processo?

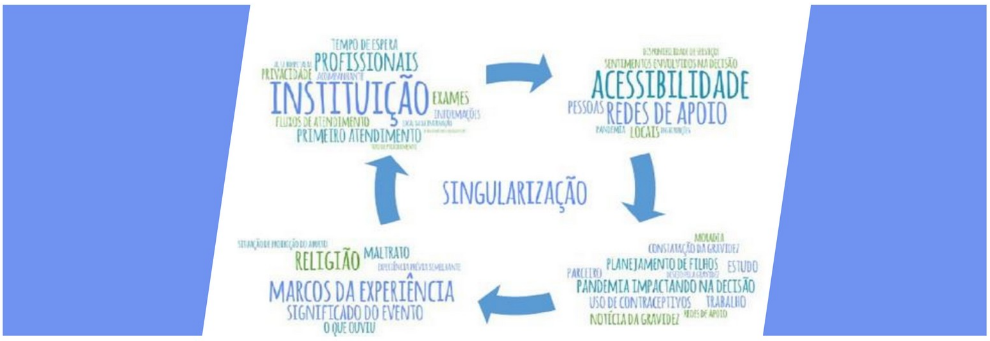

Lembre de estimular a entrevistada a descrever a experiência com o abortamento com suas próprias palavras e procure utilizar os mesmos termos, além de estabelecer um diálogo acolhedor e bem informado com a interlocutora em relação aos seus direitos como participante da pesquisa e cidadã. Informe sobre a realização de outras etapas da pesquisa e sobre onde se informar a respeito sempre que houver interesse.

Instrumento elaborado em 2020 e atualizado em 2022 por

GRUPO DE PESQUISA REPGEN
GÊNERO, REPRODUÇÃO E JUSTIÇA
